# Neonatal Heart-Enriched miR-708 Promotes Differentiation of Cardiac Progenitor Cells in Rats

**DOI:** 10.3390/ijms17060875

**Published:** 2016-06-07

**Authors:** Shengqiong Deng, Qian Zhao, Xianjin Zhou, Lin Zhang, Luer Bao, Lixiao Zhen, Yuzhen Zhang, Huimin Fan, Zhongmin Liu, Zuoren Yu

**Affiliations:** 1 Key Laboratory of Arrhythmias of the Ministry of Education of China, Research Center for Translational Medicine, Translational Medical Center for Stem Cell Therapy, East Hospital, Tongji University School of Medicine, 150 Jimo Road, Shanghai 200120, China; joan0626@126.com (S.D.); tiankong74177@126.com (Q.Z.); zhanglin1209@tongji.edu.cn (L.Zha.); baoluer@tongji.edu.cn (L.B.); zlxiao56@163.com (L.Zhe.); athero_tj@163.com (Y.Z.); frankfan@tongji.edu.cn (H.F.); 2 Sino-French Cooperative Central Laboratory, Shanghai Gongli Hospital, Second Military Medical University, Shanghai 200135, China; 3 Department of Anesthesia, Shanghai First Maternity and Infant Hospital, Tongji University School of Medicine, Shanghai 200120, China; zhouxianjin81@163.com

**Keywords:** miR-708, cardiac stem/progenitor cells, cardiomyocytes, heart regeneration

## Abstract

Cardiovascular disease is becoming the leading cause of death throughout the world. However, adult hearts have limited potential for regeneration after pathological injury, partly due to the quiescent status of stem/progenitor cells. Reactivation of cardiac stem/progenitor cells to create more myocyte progeny is one of the key steps in the regeneration of a damaged heart. In this study, miR-708 was identified to be enriched in the neonatal cardiomyocytes of rats, but this has not yet been proven in adult humans. A lower level of miR-708 in c-kit(+) stem/progenitor cells was detected compared to non-progenitors. Overexpression of miR-708 induced cardiomyocyte differentiation of cardiac stem/progenitor cells. This finding strengthened the potential of applying miRNAs in the regeneration of injured hearts, and this indicates that miR-708 could be a novel candidate for treatment of heart diseases.

## 1. Introduction

The mammalian heart has very limited regenerative capacity after pathological injury partly due to the quiescent status of stem/progenitor cells in adult hearts. During cardiac development in mammals, cardiomyocytes undergo hyperplastic to hypertrophic transition before birth. Shortly after birth, the majority of cardiomyocytes withdraw from the cell cycle and become so-called terminally differentiated cells, although there is still a low rate of cardiomyocyte turnover [1,2]. Emerging evidence indicates that the adult heart may still maintain the regenerative potential, especially under certain pathological conditions because of the existence of a small population of cells in hearts called cardiac stem cells (CSCs) or cardiac progenitor cells (CPCs).

Different groups have identified side population (SP) cells, c-kit(+) cells, Sca-1(+) cells, and Isl1(+) cells as CSCs or CPCs [3,4,5], which have stemness properties in common including self-renewal, clonogenicity and multipotency *in vitro* and *in vivo* [1]. Furthermore, they are capable of differentiating into cardiac cell types including cardiomyocytes, endothelial and smooth muscle cells [1,6,7]. Thus, CSCSs/CPCs hold great promise for maintaining cardiac cells remedying the physiological turnover of cardiomyocytes. They are one of the best potential sources for the regeneration of damaged heart and functional recovery of damaged myocardium [8]. However, the limited number and quiescent disposition of CSCSs/CPCs within adult hearts are the biggest shortage for cardiac regeneration. It has been demonstrated that CSC number increases in acute myocardial infarction [9]. Differentiation of CSCs is activated in response to ischemic injury [9].

Transplantation of various types of exogenous CSCs has been tested in clinical trials [10,11]. Cardiac c-kit(+) cells have been described as a multipotent cell population. A phase 1 trial using c-kit(+) cells showed improved left ventricle (LV) systolic function and reduced infarct size in patients with heart failure after myocardial infarction [10]. Another type of CPCs called cardiosphere-derived cells (CDCs), reduced scarring after myocardial infarction, increased viable myocardium, and boosted cardiac function in preclinical models [12]. A phase 1 clinical trial showed that patients treated with CDCs had reduction in scar mass, increase in viable heart mass and thickness in the regional systolic wall [12].

miRNAs are a class of small non-coding RNA molecules regulating the expression of targeted messenger RNAs at posttranscriptional levels [13]. More than 2000 miRNA molecules have been identified from human, mouse and/or rat tissues/cells by RNA cloning or deep sequencing [14]. miRNAs are characterized by high conservation between species and base-pairing interactions with binding site(s) of target mRNAs mostly within the 3′ untranslated region (3′UTR). miRNAs have been well demonstrated to be involved in regulation of many biological processes including embryonic development, cell division, self-renewal and differentiation of tissue stem cells, cancer initiation and progression, and cardiovascular diseases, *et al.* [15,16,17].

A few miRNAs are found to be enriched in the heart including miR-1, miR-133, miR-208a, miR-208b, and miR-499. These miRNAs have been shown to play important roles in regulating cardiac development, cardiovascular diseases, and cardiac remodeling [18]. In this study, miR-708 was identified to be abundant in the neonatal heart while the expression level markedly reduced in adult rat hearts. A lower level of miR-708 in c-kit(+) CSCs was detected compared to non-progenitors. Overexpression of miR-708 promoted differentiation of CSCs to cardiomyocytes.

## 2. Results

### 2.1. Identification of miR-708 as a Cardiomyocytes-Enriched miRNA in the Heart of Neonatal Rats

**Figure 1:**
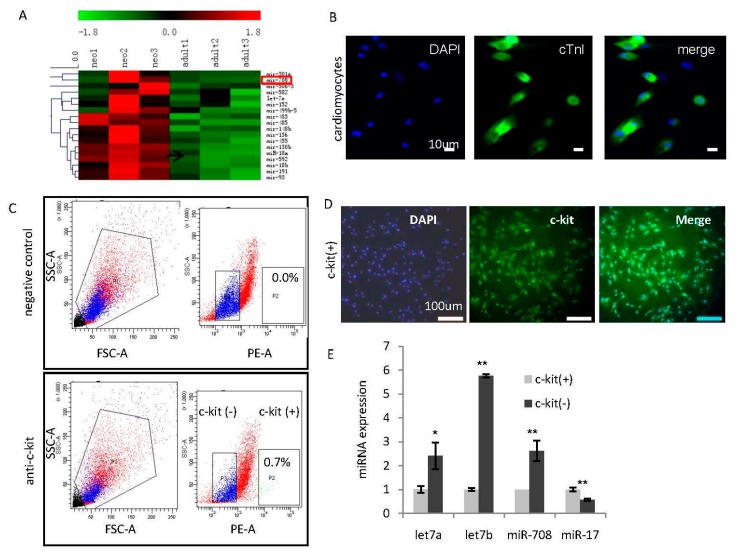
miR-708 is enriched in non-progenitor cardiomyocytes of neonatal rat. (**A**) miRNA profiling analyses between three neonatal and three adult heart tissues in rat identified a subset of miRNAs with higher expression in the neonatal hearts compared to adult hearts including miR-708 which is highlighted with red box; (**B**) Immunofluorescence staining of cardiomyocytes-specific marker cardiac troponin I (cTnI) in cardiomyocytes isolated from fresh heart tissues of neonatal rat; (**C**) FACS analysis isolated c-kit(+) cells from fresh hearts of neonatal rat; (**D**) Immunofluorescence staining of the isolated c-kit(+) cardiac stem cells (CSCs)/cardiac progenitor cells (CPCs); (**E**) A miRNA quantitative analysis demonstrated higher expression of miR-708, let-7a, let-7b and lower expression of miR-17 in c-kit(−) non-progenitor cardiomyocytes than c-kit(+) progenitors. Data are mean ± SEM (*n* = 3). * *p* < 0.05, ** *p* < 0.01.

In order to identify the key miRNAs in maintaining the active status of cardiomyocytes, miRNA profiling analyses were performed and compared inneonatal and adult heart tissues of rats. As shown in Figure 1A, a subset of neonatal hearts-enriched miRNAs including miR-708 were identified (Figure 1A). Cardiomyocytes were separated from fibroblast cells in the neonatal hearts, and further confirmed by immunofluorescence staining with cardiomyocytes-specific marker cardiac troponin I (cTnI) (Figure 1B).

It has been well demonstrated there is a small population of endogenous cardiomyocytes in the neonatal heart with c-kit positive property having progenitor cell characteristics [1]. In order to further determine the expression pattern of miR-708 in neonatal cardiomyocytes, c-kit(+) cells were purified from fresh neonatal rat hearts through cell isolation and fluorescence-activated cell sorter (FACS) analysis, and further confirmed by immunofluorescence staining (Figure 1C,D). miRNA analysis showed a higher expression of miR-708 in c-kit(−) non-progenitor cardiomyocytes than c-kit(+) progenitor cells (Figure 1E). As positive controls, differentiation miRNA let-7 showed lower expression while miR-17 showed higher expression in c-kit(+) progenitors (Figure 1E).

### 2.2. miR-708 Was Upregulated upon Differentiation of Cardiac Stem/Progenitor Cells

**Figure 2:**
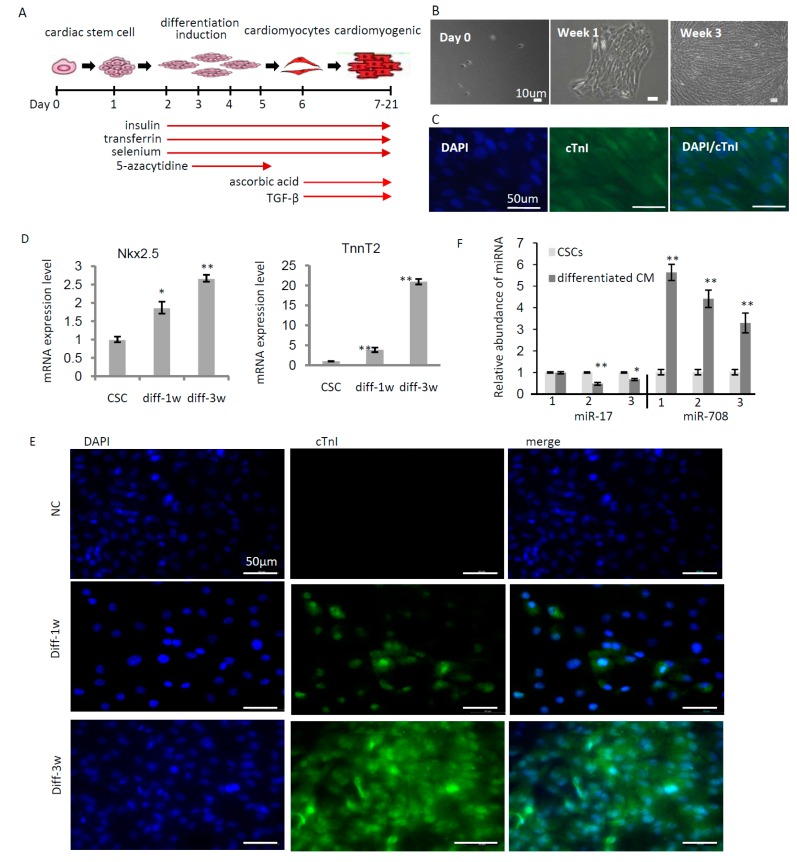
miR-708 was upregulated upon differentiation of cardiac stem/progenitor cells. (**A**) Schematic representation of the procedure for the differentiation induction of cardiac stem/progenitor cells to cardiomyocytes *in vitro*; (**B**) Representative photographs showing the differentiation of c-kit(+) cardiac progenitor cells to cardiomyocytes, forming myocardium-like strips in the cell culturing system; (**C**) Immunofluorescence staining of cardiac troponin I (cTnI) in the cardiomyocytes differentiated from cardiac progenitors; (**D**) Quantitative analysis of Troponin T Type 2 (TnnT2) and NK2 transcription factor related, locus 5 (NKX2.5) expression in cardiac stem/progenitor cells and differentiated cells after 1-week and 3-weeks of differentiation; (**E**) Immunofluorescence staining of cTnI in the differentiated cells after 1-week and 3-weeks of differentiation from cardiac progenitors. IgG as negative control (NC) for anti-cTnI; (**F**) A quantitative analysis of miR-17 and miR-708 expression in cardiac stem/progenitor cells and differentiated cells indicating the downregulation of miR-17 and upregulation of miR-708 upon differentiation. diff: differentiation. Data are mean ± SEM (*n* = 3). * *p* < 0.05, ** *p* < 0.01.

C-kit(+) CSCs/CPCs can be induced to differentiate into different cell types. The induction condition of differentiation to cardiomyocytes has been experimentally validated and reported [19] as shown in Figure 2A. Following the procedure, we successfully induced c-kit(+) cells to differentiate to cardiomyocytes in one week, and continuously cultured for an additional two weeks for cardiomyogenesis *in vitro* (Figure 2B). A cardiomyocyte marker, cardiac troponin I (cTnI), was tested by immunofluorescence staining to confirm the cardiac myogenic induction from c-kit(+) cells (Figure 2C). In addition, other cardiomyocyte markers, including cardiac Troponin T Type 2 (TnnT2) and NKX2.5, were analyzed in expression, indicating the increased levels in 1-week and 3-weeks after differentiation induction (Figure 2D). Immunofluorescence staining also confirmed the increased levels of cTnI in 1-week and 3-weeks of differentiation induction (Figure 2E). Meanwhile, the expression of miR-17 and miR-708 were analyzed before and after differentiation indicating the downregulation of miR-17 and upregulation of miR-708 upon differentiation of c-kit(+) cells (Figure 2F), suggesting that miR-708 may positively regulate differentiation of cardiac stem/progenitor cells.

### 2.3. miR-708 Promoted Differentiation of Cardiac Stem/Progenitor Cells into Cardiomyocytes

**Figure 3:**
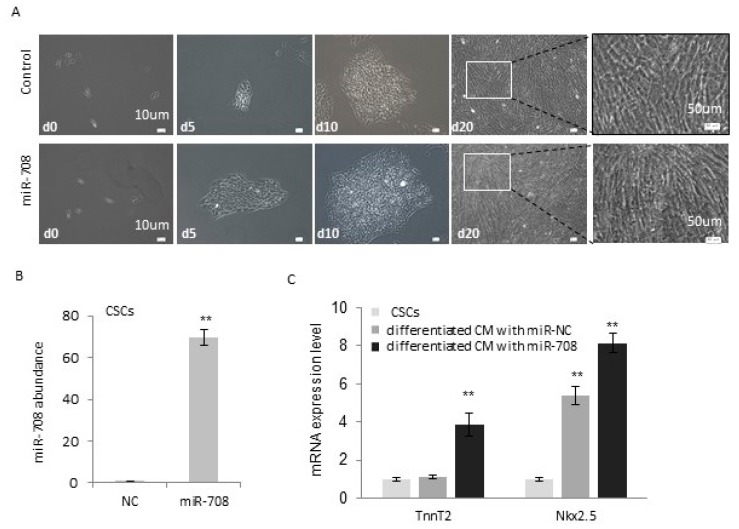
miR-708 promoted differentiation of cardiac stem/progenitor cells to cardiomyocytes. (**A**) Cardiac stem/progenitor cells were transfected with miR-708 mimics or control miRNA mimics followed by a three-week induction of differentiation; (**B**) Confirmation of miR-708 overexpression by QRT-PCR after transfection of miR-708 mimics into c-kit(+) cells; (**C**) Gene expression analysis of cardiomyocyte markers TnnT2 and NKX2.5 in c-kit(+) cardiac stem/progenitor cells before and after differentiation (one week) with or without the overexpression of miR-708. Data are mean ± SEM (*n* = 3). ** *p* < 0.01.

In order to determine the function of miR-708 during cell differentiation, the c-kit(+) cells were transfected with miR-708 or control, and cultured for ~3 weeks under the differentiation condition (Figures 2A and 3A). The overexpression of miR-708 in the c-kit(+) cells was confirmed by quantitative real-time PCR analysis (Figure 3B). miR-708 transfected cells had more capability to differentiate to cardiomyocytes and form the strips of heart muscle (Figure 3A). The gene expression analysis indicated the higher levels of cardiomyocyte markers TnnT2 and NKX2.5 after 1 week of differentiation induction in the presence of miR-708 (Figure 3C).

**Figure 4:**
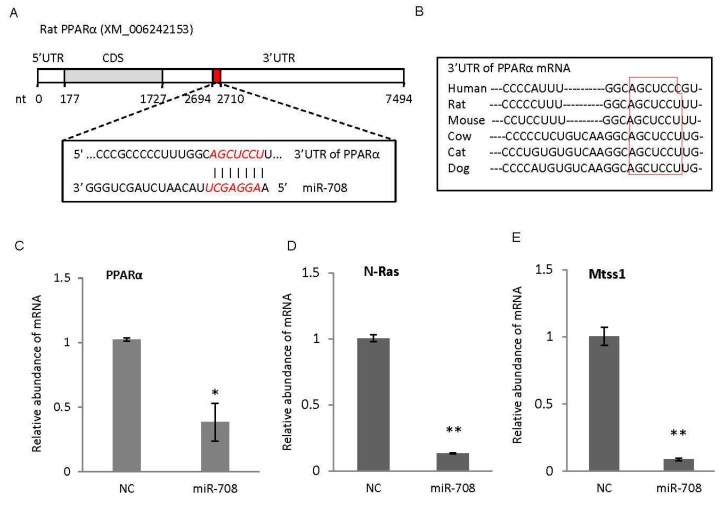
miR-708 mimics interacted with predicted target genes in cardiac stem/progenitor cells. (**A**) One binding site to miR-708 (shown in red) was identified from 3′ untranslated region (3′UTR) of peroxisome proliferator-activated receptor alpha α (PPARα) mRNA in rat; (**B**) The miR-708 binding site in PPARα mRNA is highly conserved between species as shown in red box; (**C**) Quantitative real-time PCR confirmed the decreased level of PPARα in CSCs/CPCs after transfection with miR-708; (**D**,**E**) Quantitative real-time PCR confirmed the decreased level of N-Ras (**D**) and Mtss1 (**E**) in CSCs/CPCs after transfection with miR-708. N-Ras and Mtss1 have been reported to be two target genes of miR-708 in rat. β-actin was used for normalization. Data are mean ± SEM (*n* = 3). * *p* < 0.05, ** *p* < 0.01.

In order to confirm the exogenous miR-708 is functional in cells, c-kit(+) CSCs/CPCs were transfected with miR-708 mimics. After 24 h, RNA was isolated and gene expression was analyzed for predicted target genes including PPARα (peroxisome proliferator-activated receptor alpha) which has not been reported yet, and N-Ras (N-Rasproto-oncogene) and Mtss1 (metastasissuppressor 1) which have been reported to be two target genes of miR-708 in rat [20,21]. As shown in Figure 4A,B, rat PPARα mRNA has one binding site to miR-708, which is highly conserved between species. Enforced expression of miR-708 in CSCs/CPCs was able to decrease the expression of PPARα, N-Ras and Mtss1 as shown in Figure 4C–E, indicating miR-708 mimics were able to interact with target genes in CSCs/CPCs regulating cellular differentiation, although additional works are still required to identify and confirm the target genes through which miR-708 promotes cardiomyocytes differentiation.

## 3. Discussion

In this report, we determined the differentially expressed miRNAs in neonatal hearts compared to adult rat hearts, finding the enrichment of miR-708 in neonatal cardiomyocytes and upregulation of miR-708 upon CSCs/CPCs differentiation. Overexpression, miR-708 was demonstrated to induce differentiation of CSCs/CPCs. Other data from *in vivo* assay demonstrated that local delivery of miR-708 into a mice model with injury promoted heart regeneration and increased recovery of heart function (data not shown). These findings strengthened the potential of applying miRNAs for regeneration of injured heart, and demonstrated miR-708 as a novel candidate for treatment of heart diseases.

Cardiac c-kit(+) cells have been widely reported to be a cell population with multipotency. Recently, a lineage tracing study claimed limited potential of c-kit(+) cells differentiating into cardiomyocytes during heart regeneration in mice [22]. Nishat *et al.* reported that the majority of c-kit(+) cells in the murine heart belongs to endothelial cells, while only a small number of resident c-kit(+) cells are cardiomyocytes in the tested mice [23]. In spite of the controversy, transplantation of c-kit(+)/lineage(−) CSCs was proved to improve post infarction LV dysfunction when administered to animals [10]. A phase 1 trial using this cell type showed that intracoronary infusion autologous CSCs can improve LV systolic function and reduce infarct size in patients with heart failure after myocardial infarction [10].

Cardiovascular disease is becoming the leading cause of death all over the world. Although progress has been made in the pharmacologic and device management and gene or cell therapy of heart failure, the mortality in heart failure patients remains significant. In addition to medicines, surgery sometimes prevents further damage to the heart and improves the function of the heart. An implantable left ventricular assist device (LVAD) may help patient’s heart pump blood throughout the body. When heart failure is very severe such that all other therapies are not applicable, heart transplant is usually considered. However, the limited donor sources and high risk of the surgery restrain the wide application of heart transplantation. Overall, all of these pharmacologic or surgical approaches have limited effects on the recovery of heart function. Identification of CSCs/CPCs in hearts provides a novel and promising approach for cardiac regeneration. Clinical trials transplanting exogenous CSCs/CPCs into damaged hearts for cardiac regeneration therapy [10,11] have derived promising effects, including improved LV function and reduced infarct size. However, accommodation of enough number of transplanted cells in the damaged area of hearts, differentiation induction of transplanted CSCs/CPCs to cardiomyogenic cells in recipients and functional cooperation between the CSCs/CPCs-derived myocyte progeny and endogenous cardiomyocytesare still big challenges, which impede the efficiency of CSCs/CPCs-based therapy. The current study strengthened the potential of applying miRNAs and CSCs/CPCs for heart regeneration.

## 4. Materials and Methods

### 4.1. Cells and Cell Culture

Cardiomyocytes from newborn rats was prepared by collagenase II digestion and cultured in DMEM/M199 medium with 10% FBS, 100 U/mL of penicillin, 100 μg/mL of streptomycin and 0.1 mM of Brdu (Sigma, St. Louis, MO, USA). C-kit(+) CSCs were purified by staining with PE-conjugated anti-c-kit monoclonal (#553355, BD) and sorting with a FACS Aria high-speed cell sorter (Becton Dickinson, San Jose, CA, USA), cultured in DMEM-Ham’s F-12 containing 10 ng/mL of EGF (PeproTech, Princeton, NJ, USA), 20 ng/mL of bFGF (PeproTech), 10 ng/mL of LIF (PeproTech), 10% of ES-cell qualified FBS (Gibco, Carlsbad, CA, USA), penicillin (100 U/mL) and streptomycin (100 μg/mL). The cardio fibroblasts were removed from cardiomyocytes through differential adhesion.

### 4.2. Oligos and Transfection

All primers, miR-708 mimic and negative control oligo were synthesized by GenScript (Nanjing, China). Primer sequences for mRNAs are available upon request. Forward primer sequences for real-time PCR of miRNAs were: miR-708: 5′-GAGCUUACAAUCUAGCUG-3′, miR-17-5p: 5′-CAAAGTGCTTACAGTGC-3′, and 5s rRNA: 5′-AGTACTTGGATGGGAGACCG-3′. The miRNA mimic sequences for miR-708: 5′-AAGGAGCUUACAAUCUAGCUGGG-3′. The HiPerFect transfection reagent from Qiagen (Venlo, The Netherland) was used for cell transfection following the manufacturer’s instructions. Final concentration of 30 nM of miRNA mimics was used for all *in vitro* assays.

### 4.3. miRNA QRT-PCR Analysis

A M&G miRNA Reverse Transcription Kit (miRGenes, Shanghai, China) was used to synthesize the first strand cDNA of miRNA as described before [24]. ABI 7900 HT and ABI 7500 Sequence Detection System (PE Applied Biosystems, Foster City, CA, USA) were used for Quantitative Real Time PCR analysis of miRNAs and 5s rRNA was used for normalization.

### 4.4. miRNA Profiling Analysis in Neonatal and Adult Rat Heart

Total RNA was isolated from plasma specimens with Trizol reagent (Life Technologies, New York, NY, USA). The cDNA was prepared as described above. The miRNA profiling analyses were performed with the quantitative real-time PCR based miRNA panel which contains 365 miRNAs and five reference small RNAs (miRGenes, Shanghai, China). The data was analyzed by Mev (version 4.9, Boston, MA, USA) software. *p* < 0.05 was considered as significant.

### 4.5. Immunofluorescence

Cells were cultured in 6-well plates with cell-attaching slides in the wells. Before immunofluorescenceanalysis, cells on the slides were fixed with 4% paraformaldehyde for 15 min, permeabilized with 0.1% Triton X-100 and then blocked with 1% BSA for 1 h. After incubation with primary antibody (1:10 to 1:100 dilution) overnight at 4 °C, FITC-conjugated goat anti rabbit IgG (ab6717, Abcam, Cambridge, UK, 1:200 dilution) was used as secondary antibody. 6-diamidino-2-phenylindole (DAPI) was used for nuclear counterstaining. The slides were photographed using fluorescence microscopy (Leica, Mannheim, Germany). Primary antibodies: anti-cTnI (ab47003, Abcam); anti-c-kit (#H-300, Santa Cruz, CA, USA). All immunofluorescenceanalyses were performed independently three times.

### 4.6. Induced Differentiation of CSCs

The method for differentiation induction of CSCs/CPCs to cardiomyocytes has been described previously [19] and showed in Figure 2A. Briefly, CSCs were seeded at a density of ~10,000 cells/cm^2^. The differentiation medium containing 50% IMDM and 50% Ham’s F12 nutrient mixture with GlutaMAX-I, 2% horse serum, 1× MEM nonessential amino acids, 1× insulin (10 mg/L)–transferring (5.5 mg/L)–selenium (67 μg/L), penicillin (100 U/mL) and streptomycin (100 μg/mL), was applied the next day. From day 3 to day 5, 5 μM 5-azacytidine (A2385, Sigma) was applied. From day 6 on, the cells were treated with 0.1 mM ascorbic acid (A4403, Sigma) every 2 days and 1 ng/mL TGF-β (#100-21C, PeproTech) twice weekly.

### 4.7. Statistical Analysis

Data are presented as mean ± SEM. The standard two-tailed student’s *t*-test was used for statistical analysis, in which *p* < 0.05 was considered significant.

## 5. Conclusions

miR-708 is enriched in the neonatal cardiomyocytes of rats. Overexpression of miR-708 is able to promote cardiomyocyte differentiation of cardiac stem/progenitor cells.
